# Impact of Prosthetic Rehabilitation on Oral Health-Related Quality of Life in Patients With Oral Cancer at a Tertiary Care Center in Kerala

**DOI:** 10.7759/cureus.96454

**Published:** 2025-11-09

**Authors:** Geethu R M, Smitha L Rajeev, Vivek V Nair, Prasanth Viswambharan

**Affiliations:** 1 Department of Prosthodontics, Government Dental College, Thiruvananthapuram, Thiruvananthapuram, IND

**Keywords:** oral health, oropharyngeal cancer, prosthetic rehabilitation, psychological well-being, quality of life, reconstructive surgery, social isolation

## Abstract

Background: Negative physical and psychological impacts may result from oral cancer and its treatment, causing social isolation and a decreased quality of life. Restoring the patient's functional and psychological well-being following reconstructive surgery requires prosthetic rehabilitation.

Aim: This study aimed to determine the effect of prosthetic rehabilitation on Oral Health-Related Quality of Life (OHRQoL) in patients with oral cancer, comparing pre- and postrehabilitation statuses.

Methods and materials: Forty-four patients who met the inclusion criteria of having completed treatment for various oral cancers and requiring prosthetic rehabilitation, with an age range of 20-75 years, were selected for the study. Using Liverpool Oral Rehabilitation Questionnaire version 3 (LORQv3) and Oral Health Impact Profile (OHIP)-14, study participants completed a self-report survey questionnaire on their experiences with oral problems before rehabilitation and at one month and six months postrehabilitation.

Statistical analysis: Normality was assessed, and comparisons of outcome variables were performed using repeated measures analysis of variance. A correlation analysis was performed between related continuous variables using the Pearson correlation test.

Results and conclusion: A noticeable improvement was observed in almost all domains of LORQv3 and OHIP-14, across both follow-ups. Regarding oral function, the LORQv3 showed improvement in chewing (3.39 ± 0.78 → 2.24 ± 0.60 → 1.54 ± 0.44, η² = 0.874) and swallowing (3.48 ± 0.76 → 1.96 ± 0.60 → 1.32 ± 0.41, η² = 0.879) from prerehabilitation to one and six months postrehabilitation, respectively. Similarly, OHIP-14 domains of functional limitation (3.09 ± 0.75 → 1.05 ± 0.81 → 0.50 ± 0.72, η² = 0.854) and physical disability (3.00 ± 0.74 → 1.06 ± 0.75 → 0.46 ± 0.58, η² = 0.841) reflected comparable improvements. In terms of comfort and physical well-being, LORQv3 salivation (2.65 ± 0.84 → 1.70 ± 0.44 → 1.29 ± 0.33, η² = 0.703) and mouth opening (2.77 ± 0.96 → 1.77 ± 0.74 → 1.36 ± 0.48, η² = 0.708) scores decreased steadily, aligning with OHIP-14 physical pain (3.17 ± 0.65 → 1.13 ± 0.87 → 0.51 ± 0.75, η² = 0.847), indicating reduced oral discomfort. Improvements in speech, appearance, and psychosocial domains were also evident, with LORQv3 speech (3.36 ± 0.74 → 2.06 ± 0.78 → 1.52 ± 0.62, η² = 0.782), orofacial appearance (3.18 ± 0.88 → 2.09 ± 0.72 → 1.67 ± 0.74, η² = 0.728), and social interaction (3.31 ± 0.90 → 2.13 ± 0.66 → 1.65 ± 0.74, η² = 0.758) showing consistent enhancement. Correspondingly, OHIP-14 domains such as psychological discomfort (2.92 ± 0.81 → 1.21 ± 1.08 → 0.61 ± 0.93, η² = 0.775), social disability (3.27 ± 0.81 → 2.15 ± 1.30 → 1.82 ± 1.44, η² = 0.482), and handicap (3.97 ± 0.67 → 2.69 ± 1.13 → 2.01 ± 1.15, η² = 0.788) demonstrated progressive improvement. Overall, both LORQv3 and OHIP-14 revealed parallel trends with mean score reductions of approximately 1.5-2.5 points across overlapping domains, confirming significant enhancement in oral function, comfort, and psychosocial quality of life following rehabilitation. Gender, economic status, geography, and treatment type significantly influenced outcomes of LORQv3 and OHIP-14. Strong correlations between domains of OHIP-14 and LORQv3 were observed. These results highlight the importance of timely, interdisciplinary prosthetic rehabilitation, which not only facilitates functional restoration but also greatly improves OHRQoL.

## Introduction

Oral cancer patients often undergo a combination of radiation, surgery, and chemotherapy that significantly alters the social, mental, and physical welfare of affected individuals. Substantial psychological morbidity may be secondary to postoperative facial deformation as well as functional deficits in speaking, deglutition, and mastication. Negative physical and psychological impacts may result from neoplasms in the oral cavity and related treatment, causing isolation from society and a decreased standard of living [1]. These issues have been somewhat mitigated by well-established and advancing microvascular reconstructive procedures for orofacial rehabilitation. Good orofacial function and esthetics can be achieved with modern reconstructive procedures, although neither is guaranteed. Restoring the patient's functional and psychological well-being following reconstructive surgery requires prosthetic rehabilitation. According to Dalkiz and Dalkiz, early prosthetic intervention after oral cancer resection improves speech intelligibility, swallowing efficiency, and psychosocial outcomes [2]. At the time of diagnosis of oral cancer, survival becomes the primary concern for the patient [3]. Several studies have shown that patients' physical and emotional functioning, as well as their general health state, are at their lowest during the diagnosis and the first few months after treatment [3-5]. However, most patients' quality-of-life scores improve by the end of the first year. The majority of the patients continue to fight issues with facial deformities, emotional and cognitive malfunctioning, and chewing-related problems such as sticky saliva, dry mouth, and chronic eating difficulties, when their focus shifts from surviving to preserving and enhancing their way of life. Quality of life as defined by the WHO is ‘‘an individual’s perception of their own position in life, in the context of the culture and value systems in their life and in relation to their goals, expectations, standards and concerns” [4]. Three approaches to evaluating Health-Related Quality of Life (HRQoL) are unstructured interviews, semistructured interviews, and patient-reported questionnaires, with patient-reported questionnaires being the most exceptional [5]. HRQoL questionnaires help to document outcomes, guide treatment decisions, and support posttreatment interventions. Understanding the changes in oral health-related quality of life (OHRQoL) before and after prosthetic rehabilitation is crucial for evaluating the functional and psychosocial benefits of rehabilitation, identifying persistent unmet needs, and informing strategies for improving long-term outcomes among oral cancer survivors. Despite substantial international evidence on the impact of prosthetic rehabilitation on OHRQoL in oral cancer survivors, longitudinal data from the Indian population, integrating both tools to provide a comprehensive assessment of functional and psychosocial recovery, remain limited, particularly from South Indian tertiary care centers. Furthermore, the long-term outcomes of maxillofacial prosthetic rehabilitation, especially in relation to speech, mastication, and social reintegration, remain underreported in regional populations. Therefore, it is vital to understand the quality of life connected to oral health during the prosthetic rehabilitation phase and how it changes over the postrehabilitation period.

## Materials and methods

Study design and setting

This observational longitudinal study was conducted on patients who had completed management for various neoplasms of the oral cavity and required prosthetic rehabilitation. The study adhered to the Strengthening the Reporting of OBservational studies in Epidemiology guidelines for observational research reporting.

Participants

A total of 44 patients, aged 20-75 years, who had completed management for different neoplasms in the oral cavity and required a prosthesis, were selected for the study.

Inclusion and exclusion criteria

Participants were selected irrespective of the site, its histopathology, and mode of treatment. Patients who were not willing to give consent, uncooperative patients, those having severe complications like trismus, and patients possessing substantial defects, which narrowed the possibility of a successful rehabilitation, were excluded from the study.

Data collection and instruments

Research participants were asked to evaluate their oral condition and problems before rehabilitation, and then follow up at one and six months after rehabilitation using two questionnaires: the Liverpool Oral Rehabilitation Questionnaire version 3 (LORQv3) and the Oral Health Impact Profile (OHIP)-14 (see the Appendix). The main validated questionnaires for assessing OHRQoL include the Oral Health Impact Profile (OHIP), Oral Impacts on Daily Performance, Geriatric Oral Health Assessment Index, European Organization for Research and Treatment of Cancer Quality of Life Questionnaire, Dental Impact on Daily Living, OHRQoL Scale, and the LORQv3 [6]. Among these, LORQv3, specifically designed for patients undergoing oral or maxillofacial prosthetic rehabilitation, was selected for its specificity, sensitivity, and comprehensive patient-centered assessment, while OHIP-14 was chosen for its efficiency, global comparability, and strong theoretical foundation. The LORQv3 consists of 40 items divided into two sections. The first 17 items assess issues related to oral function, social interaction, and orofacial appearance. The rest deal with prosthesis and patient denture/prosthetic satisfaction. Patient responses were rated on a Likert scale with scores ranging from 1 to 4, where 1 = never, 2 = sometimes, 3 = often, and 4 = always [7,8].

The OHIP-14 assesses functional limitation, physical pain, psychological discomfort, physical disability, psychological disability, social disability, and handicap. Patient experiences were noted on a Likert Scale as Never = 0, Hardly Ever = 1, Sometimes = 2, Often = 3, and Very Often = 4 [9]. The content validity of the LORQv3 and OHIP-14 questionnaires was assessed during the course of the study.

Statistical analysis

IBM Statistical Package for the Social Sciences version 26 (IBM Corp., Armonk, NY) for Windows was used to code, tabulate, and analyze observed data. Descriptive statistics for continuous variables were reported as mean and standard deviation, and categorical variables were reported as percentages and frequencies. Normality was assessed, and repeated measures analysis of variance was used. Correlation between related continuous variables was determined using the Pearson correlation test. A p value of less than 0.05 was considered statistically significant.

## Results

Out of the 44 participants, 28 (63.60%) were men and 16 (36.40%) were women, with an age range of 27-73 years (mean = 54 years). They were rehabilitated with obturators. Twenty-two (50%) participants fell above the poverty line, while the rest fell below it. Twenty-eight (63.60%) belong to the rural area. The most common type of malignancy was squamous cell carcinoma, 35 (79.50%), and the most common type of treatment was a combination of surgery, chemotherapy, and radiotherapy, 19(43.2%). Table 1 presents a comparison of the mean scores of LORQv3 domains at baseline and at the first and second follow-ups. Every domain of LORQv3, except mandibular patient satisfaction, demonstrated a statistically significant difference (p = 0.095).

**Table 1:** Comparison of mean scores of LORQv3 domains: prerehabilitation, postrehabilitation after one month, and postrehabilitation phase after six months ^*^A p value of <0.05 was found to be significant LORQv3: Liverpool Oral Rehabilitation Questionnaire version 3; PR: prerehabilitation; PO1: postrehabilitation after one month; PO2: postrehabilitation after six months; SD: standard deviation

LORQv3 domains	n	PR (mean ± SD)	PO1 (mean ± SD)	PO2 (mean ± SD)	Percentage change scores from PR to PO1	Percentage change scores from PR to PO1	p value
Chewing (1,2,16)	44	3.39 ± 0.78	2.24 ± 0.60	1.54 ± 0.44	33.03	29.82	<0.001^*^
Swallowing (3,4)	44	3.48 ± 0.76	1.96 ± 0.60	1.32 ± 0.41	42.10	27.12	<0.001^*^
Salivation (5-9)	44	2.65 ± 0.84	1.70 ± 0.44	1.29 ± 0.33	31.58	22.06	<0.001^*^
Speech (10)	44	3.36 ± 0.74	2.06 ± 0.78	1.52 ± 0.62	38.25	22.15	<0.001^*^
Mouth opening (17)	44	2.77 ± 0.96	1.77 ± 0.74	1.36 ± 0.48	33.33	17.04	<0.001^*^
Orofacial appearance (11-14)	44	3.18 ± 0.88	2.09 ± 0.72	1.67 ± 0.74	32.34	19.09	<0.001^*^
Social interaction (15)	44	3.31 ± 0.90	2.13 ± 0.66	1.65 ± 0.74	33.14	21.59	<0.001^*^
Patient satisfaction (20-25)	44	-	1.49 ± 0.12	1.40 ± 0.10	-	5.87	<0.001^*^
Maxillary patient satisfaction (26-31)	44	-	1.96 ± 0.57	1.53 ± 0.54	-	21.85	<0.001^*^
Mandibular patient satisfaction (34-39)	44	-	1.94 ± 0.19	1.44 ± 0.48	-	26.80	0.095

Patients perceived an improvement in chewing, 14 (33.03%), swallowing, 18 (42.10%), salivation, 13 (31.58%), speech, 16 (38.25%), mouth opening, 14 (33.33%), orofacial appearance, 13 (32.34%), and social interaction, 14 (33.14%), from baseline to the first follow-up. Also, a betterment was seen in chewing, 12 (29.82%), swallowing, 11 (27.12%), salivation, 10 (22.06%), speech, 10 (22.15%), mouth opening, 7 (17.04%), orofacial appearance, 8 (19.09%), and social interaction, 10 (21.59%), from first to second follow-up. Maxillary and mandibular patient satisfaction rates were 10 (21.85%) and 11 (26.80%), respectively, from the first to the second follow-up, as none of them had received a prosthesis before rehabilitation.

Table 2 presents the comparison of mean scores for OHIP-14 domains at baseline and the first and second follow-ups. An amelioration was seen in functional limitation, 29 (67%), patient satisfaction, 28 (65%), psychological discomfort, 26 (60%), physical discomfort, 27 (62.40%), social disability, 15 (34%), handicap, 14 (31.75%), from baseline to the first follow-up, from first to second follow-up an improvement of functional limitation, 23 (53.50%), patient satisfaction, 28 (64.70%), psychological discomfort, 24 (56.70%), physical discomfort, 26 (60.40%), psychological disability, 26 (60%), social disability, 7 (17.60%), handicap, 11 (24.30%), was observed. All the domains of OHIP-14 revealed statistically marked variation.

**Table 2:** Comparison of mean scores of OHIP-14 domains: prerehabilitation, postrehabilitation after one month, and postrehabilitation phase after six months ^*^A p value of <0.05 was found to be significant OHIP-14: Oral Health Impact Profile 14; PR: prerehabilitation; PO1: postrehabilitation after one month; PO2: postrehabilitation after six months; SD: standard deviation

OHIP-14 domains	n	PR (mean ± SD)	PO1 (mean ± SD)	PO2 (mean ± SD)	Percentage change scores from PR to PO1	Percentage change scores from PR to PO1	p value
Functional limitation (1,2)	44	3.09 ± 0.75	1.05 ± 0.81	0.50 ± 0.72	67.23	53.50	<0.001^*^
Physical pain (3,4)	44	3.17 ± 0.65	1.13 ± 0.87	0.51 ± 0.75	65.09	64.79	<0.001^*^
Psychological discomfort (5,6)	44	2.92 ± 0.81	1.21 ± 1.08	0.61 ± 0.93	60.23	56.69	<0.001^*^
Physical disability (7,8)	44	3.00 ± 0.74	1.06 ± 0.75	0.46 ± 0.58	62.40	60.37	<0.001^*^
Psychological disability (9,10)	44	1.07 ± 0.86	1.07 ± 0.86	0.52 ± 0.77	60.00	57.90	<0.001^*^
Social disability (11,12)	44	3.27 ± 0.81	2.15 ± 1.30	1.82 ± 1.44	34.12	17.61	<0.001^*^
Handicap (13,14)	44	3.97 ± 0.67	2.69 ± 1.13	2.01 ± 1.15	31.75	24.30	<0.001^*^

**Figure 1:**
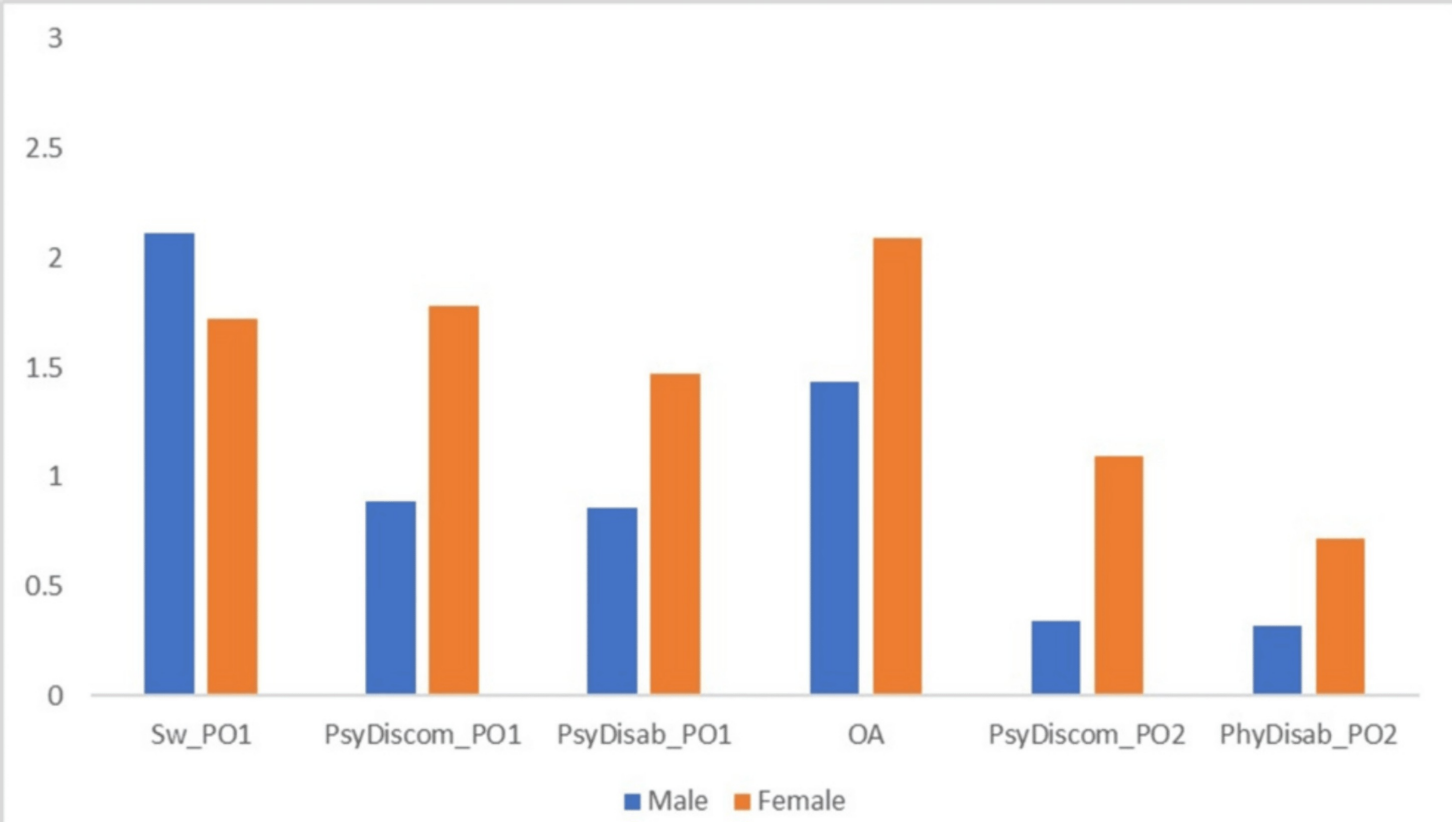
Comparison of mean OHIP-14 and LORQv3 domains based on gender OHIP-14: Oral Health Impact Profile 14; LORQv3: Liverpool Oral Rehabilitation Questionnaire version 3; Sw_PO1: swallowing at postrehabilitation phase after one month; PsyDiscom_PO1: psychological discomfort at postrehabilitation phase after one month; PhyDisab_PO1: physical disability at postrehabilitation phase after one month; OA: orofacial appearance; PsyDiscom_PO2: psychological discomfort at postrehabilitation phase after two month; PhyDisab_PO2: physical disability at post-rehabilitation phase after two month

Comparison of mean OHIP-14 and LORQv3 domains based on gender (Figure 1) revealed statistically significant differences in female participants for psychological disability (p = 0.02), psychological discomfort (p = 0.007), and swallowing (p = 0.04) at one-month follow-up, and psychological discomfort (p = 0.009), physical disability (p = 0.03), and orofacial appearance (p = 0.003) at six months follow-up.

An analysis of the mean OHIP-14 and LORQv3 scores across different economic groups showed statistically significant differences in several areas. At the one-month follow-up, significant differences were observed in psychological disability (p = 0.007), psychological discomfort (p = 0.02), and swallowing difficulties (p = 0.04). At the six-month follow-up, significant differences were noted in psychological discomfort (p = 0.009), physical disability (p = 0.03), handicap (p = 0.02), and orofacial appearance (p = 0.003) among patients in BPL patients.

Assessment of mean OHIP-14 and LORQv3 scores across the geographical area revealed a statistically significant difference among urban residents in psychological disability (p = 0.02), chewing (p = 0.04), and speech (p = 0.03) at both baseline and one-month follow-up. Upon a six-month evaluation, urban residents showed significantly higher levels of physical pain, psychological discomfort, psychological disability, physical disability (p = 0.045, 0.009, 0.006, 0.013), chewing (p = 0.010), orofacial appearance (p = 0.001), and social interaction (p = 0.02).

Performance metrics across various treatment modalities in the study cohort revealed no significant differences in OHIP-14 and LORQv3 outcomes during the preprosthetic phase. However, after one month, there were significantly higher levels of physical pain (p = 0.02) and psychological discomfort (p = 0.006), as well as lower satisfaction with maxillary prostheses (p = 0.005) among patients. Additionally, a statistically significant difference persisted in psychological discomfort (p = 0.036), orofacial appearance (p = 0.003), and satisfaction with maxillary prostheses (p = 0.002) in patients who underwent surgical treatment alone. Regression analysis (Tables 3-5) revealed significant associations between multiple domains of the OHIP-14 and LORQv3, indicating interrelated aspects of OHRQoL.

**Table 3:** Correlation between domains at prerehabilitation phase ^*^Mild correlation ^**^Moderate correlation LORQv3: Liverpool Oral Rehabilitation Questionnaire version 3; OHIP-14: Oral Health Impact Profile 14; FL_PR: functional limitation at prerehabilitation phase; PP_PR: physical pain at prerehabilitation phase; PsyDiscom_PR: psychological discomfort at prerehabilitation phase; PhyDisab_PR: physical disability at prerehabilitation phase; PsyDisab_PR: psychological disability at prerehabilitation phase; SD_PR: social disability at prerehabilitation phase; HC_PR: handicap at prerehabilitation phase; Ch_PR: chewing at prerehabilitation phase; Sw_PR: swallowing at prerehabilitation phase; Sal_PR: salivation at prerehabilitation phase; Sp_PR: speech at prerehabilitation phase; MO_PR: mouth opening at prerehabilitation phase; OA_PR: orofacial appearance at prerehabilitation phase; So_PR: social interaction at prerehabilitation phase; r: Pearson's correlation coefficient; n: sample size

Domains of LORQv3 and OHIP-14		Ch_PR	Sw_PR	Sal_PR	Sp_PR	MO_PR	OA_PR	So_PR
FL_PR	r	0.36^*^	0.20	0.04	0.39^**^	0.26	0.34^*^	0.36^*^
	p	0.01	0.18	0.76	0.009	0.07	0.02	0.02
	n	44	44	44	44	44	44	44
PP_PR	r	0.45^**^	0.27	0.04	0.43^**^	0.32^*^	0.35^*^	0.45^**^
	p	0.002	0.07	0.76	0.003	0.03	0.02	0.002
	n	44	44	44	44	44	44	44
PsyDiscom_PR	r	0.34^*^	0.23	0.40^**^	0.16	0.08	0.25	0.30^*^
	p	0.02	0.13	0.007	0.29	0.60	0.09	0.04
	n	44	44	44	44	44	44	44
PhyDisab_PR	r	0.32^*^	0.29	0.09	0.33^*^	0.13	0.28	0.30^*^
	p	0.03	0.05	0.52	0.02	0.40	0.06	0.04
	n	44	44	44	44	44	44	44
PsyDisab_PR	r	0.29^*^	0.02	0.32^*^	0.26	0.30^*^	0.27	0.24
	p	0.05	0.90	0.03	0.08	0.04	0.06	0.10
	n	44	44	44	44	44	44	44
SD_PR	r	0.52^**^	0.42^**^	0.23	0.36^*^	0.09	0.26	0.43^**^
	p	0.00	0.004	0.13	0.01	0.53	0.08	0.003
	n	44	44	44	44	44	44	44
HC_PR	r	036^*^	0.17	0.14	0.29	0.11	0.42^**^	0.41^**^
	p	0.012	0.31	0.35	0.05	0.45	0.04	0.006
	n	44	44	44	44	44	44	44

**Table 4:** Correlation between domains at postrehabilitation phase after one month ^*^Mild correlation ^**^Moderate correlation LORQv3: Liverpool Oral Rehabilitation Questionnaire version 3; OHIP-14: Oral Health Impact Profile 14; FL_PO1: functional limitation at postrehabilitation phase after one month; PP_PO1: physical pain at postrehabilitation phase after one month; PsyDiscom_PO1: psychological discomfort at postrehabilitation phase after one month; PhyDisab_PO1: physical disability at postrehabilitation phase after one month; PsyDisab_PO1: psychological disability at postrehabilitation phase after one month; SD_PO1: social disability at postrehabilitation phase after one month; HC_PO1: handicap at postrehabilitation phase after one month; Ch_PO1: chewing at postrehabilitation phase after one month; Sw_PO1: swallowing at postrehabilitation phase after one month; Sal_PO1: salivation at postrehabilitation phase after one month; Sp_PO1: speech at postrehabilitation phase after one month; MO_PO1: mouth opening at postrehabilitation phase after one month; OA_PO1: orofacial appearance at postrehabilitation phase after one month; So_PO1: social interaction at postrehabilitation phase after one month; MaxPS_PO1: patient satisfaction (maxillary) at postrehabilitation phase after one month; MandPS_PO1: patient satisfaction (mandibular) at postrehabilitation phase after one month; r: Pearson's correlation coefficient

Domains of LORQv3 and OHIP-14		Ch_PO1	Sw_PO1	Sal_PO1	Sp_PO1	MO_PO1	OA_PO1	So_PO1	PS_PO1	MaxPS_PO1	MandPS_PO1
FL_PO1	r	0.41^**^	0.47^**^	0.31^*^	0.62^**^	0.15	0.34^*^	0.19	0.35^*^	0.57^**^	0.75
	p	0.006	0.001	0.04	0.00	0.31	0.02	0.19	0.02	0.00	0.45
	n	44	44	44	44	44	44	44	44	44	3
PP_PO1	r	0.28	0.45^**^	0.30^*^	0.54^**^	0.01	0.33^*^	0.16	0.47^**^	0.63^**^	1.00^**^
	p	0.06	0.002	0.05	0.00	0.93	0.02	0.28	0.001	0.00	0.00
	n	44	44	44	44	44	44	44	44	44	3
PsyDiscom_PO1	r	0.21	0.12	0.18	0.21	0.06	0.46^*^	0.41^**^	0.26	0.59^**^	0.95
	p	0.16	0.45	0.22	0.16	0.66	0.002	0.006	0.08	0.000	0.210
	n	44	44	44	44	44	44	44	44	44	3
PhyDisab_PO1	r	0.10	0.06	0.06	0.15	0.18	0.16	0.16	0.23	0.51^**^	0.00
	p	0.52	0.72	0.69	0.33	0.24	0.28	0.28	0.14	0.000	0.000
	n	44	44	44	44	44	44	44	44	44	3
PsyDisab_PO1	r	0.36^*^	0.29	0.46^**^	0.04^*^	0.19	0.52^**^	0.34^*^	0.37^*^	0.71^**^	1.00^**^
	p	0.02	0.05	0.001	0.01	0.21	0.000	0.02	0.01	0.000	0.000
	n	44	44	44	44	44	44	44	44	44	3
SD_PO1	r	0.37^*^	0.35^*^	0.12	0.46^**^	0.06	0.42^**^	0.42^**^	0.32^*^	0.53^**^	0.36
	p	0.01	0.02	0.44	0.001	0.68	0.005	0.005	0.04	0.000	0.76
	n	44	44	44	44	44	44	44	44	44	3
HC_PO1	r	0.37^*^	0.16	0.13	0.37^*^	0.14	0.49^**^	0.43^**^	0.35^*^	0.52^**^	0.86
	p	0.01	0.29	0.42	0.01	0.38	0.001	0.003	0.02	0.000	0.33
	n	44	44	44	44	44	44	44	44	44	3

**Table 5:** Correlation between domains at postrehabilitation phase after six months ^*^Mild correlation ^**^Moderate correlation LORQv3: Liverpool Oral Rehabilitation Questionnaire version 3; OHIP-14: Oral Health Impact Profile 14; FL_PO2: functional limitation at postrehabilitation phase after six months; PP_PO2: physical pain at postrehabilitation phase after six months; PsyDiscom_PO2: psychological discomfort at postrehabilitation phase after six months; PhyDisab_PO2: physical disability at postrehabilitation phase after six months; PsyDisab_PO2: psychological disability at postrehabilitation phase after six months; SD_PO2: social disability at postrehabilitation phase after six months; HC_PO2: handicap at postrehabilitation phase after six months; Ch_PO2: chewing at postrehabilitation phase after six months; Sw_PO2: swallowing at postrehabilitation phase after six months; Sal_PO2: salivation at postrehabilitation phase after six months; Sp_PO2: speech at postrehabilitation phase after six months; MO_PO2: mouth opening at postrehabilitation phase after six months; OA_PO2: orofacial appearance at postrehabilitation phase after six months; So_PO2: social interaction at postrehabilitation phase after six months; MaxPS_PO2: patient satisfaction (maxillary) at postrehabilitation phase after six months; MandPS_PO2: patient satisfaction (mandibular) at postrehabilitation phase after six months; r: Pearson's correlation coefficient

Domains of LORQv3 and OHIP-14		Ch_PO2	Sw_PO2	Sal_PO2	Sp_PO2	MO_PO2	OA_PO2	So_PO2	PS_PO2	MaxPS_PO2	MandPS_PO2
FL_PO2	r	0.45^**^	0.28	0.55^**^	0.43^**^	0.06	0.61^**^	0.41^**^	0.29	0.69^**^	1.00^**^
	p	0.002	0.05	0.000	0.003	0.67	0.000	0.006	0.05	0.000	0.000
	n	44	44	44	44	44	44	44	44	44	3
PP_PO2	r	0.54^**^	0.39^**^	0.68^**^	0.47^**^	0.21	0.59^**^	0.39^**^	0.50^**^	0.77^**^	0.98**
	p	0.000	0.008	0.000	0.001	0.17	0.000	0.007	0.001	0.000	0.12
	n	44	44	44	44	44	44	44	44	44	3
PsyDiscom_PO2	r	0.38^*^	0.006	0.45^**^	0.09	0.08	0.81^**^	0.75^**^	0.07	0.74^**^	0.99
	p	0.01	0.96	0.002	0.54	0.58	0.000	0.000	0.635	0.000	0.07
	n	44	44	44	44	44	44	44	44	44	3
PhyDisab_PO2	r	0.62^**^	0.14	0.38^*^	0.34^*^	0.004	0.69^**^	0.56^**^	0.22	0.65^**^	0.000
	p	0.000	0.35	0.01	0.03	0.98	0.000	0.000	0.15	0.000	1.00
	n	44	44	44	44	44	44	44	44	44	3
PsyDisab_PO2	r	0.34^*^	0.06	0.41^**^	0.19	0.28	0.68^**^	0.56^**^	0.18	0.58^**^	0.97
	p	0.02	0.67	0.006	0.22	0.06	0.000	0.000	0.23	0.000	0.15
	n	44	44	44	44	44	44	44	44	44	3
SD_PO2	r	0.48^**^	0.17	0.37^*^	0.32^*^	0.02	0.56^**^	0.57^**^	0.16	0.65^**^	0.36
	p	0.001	0.26	0.01	0.04	0.87	0.000	0.000	0.29	0.000	0.76
	n	44	44	44	44	44	44	44	44	44	3
HC_PO2	r	0.49^**^	0.02	0.45^**^	0.34^*^	0.01	0.76^**^	0.64^**^	0.19	0.68^**^	0.98
	p	0.001	0.92	0.002	0.022	0.93	0.000	0.000	0.21	0.000	0.12
	n	44	44	44	44	44	44	44	44	44	3

Speech showed a positive correlation with functional limitation (p = 0.009, r = 0.39; p = 0.001, r = 0.62; p = 0.003, r = 0.43) and physical pain (p = 0.003, r = 0.43; p = 0.000, r = 0.54; p = 0.001, r = 0.47) in pre- and postrehabilitation phases at one and six months. Orofacial appearance showed a positive correlation with functional limitation, physical pain, and handicap (p = 0.02, r = 0.34; p = 0.01, r = 0.35; p = 0.004, r = 0.42) in the prerehabilitation phase and with all the domains of OHIP-14 after rehabilitation. Social interaction demonstrated a positive correlation with psychological discomfort, social discomfort, and handicap during the prerehabilitation phase (p = 0.04, r = 0.30; p = 0.003, r = 0.43; p = 0.006, r = 0.41) also at one-month (p = 0.006, r = 0.41; p = 0.005, r = 0.41; p = 0.003, r = 0.43) and six-month follow-up (p = 0.000, r = 0.75; p = 0.006, r = 0.41; p = 0.000, r = 0.57). A positive association was observed between patient satisfaction and physical discomfort (p = 0.001, r = 0.47; p = 0.001, r = 0.50) in the postrehabilitation period. Both postrehabilitation phases exhibited a positive correlation of maxillary denture satisfaction with all domains of OHIP-14, while mandibular denture satisfaction was positively correlated with only physical pain (p = 0.00, r = 1.00; p = 0.12, r = 0.98).

## Discussion

Principal findings and comparison with other studies

Patient-reported outcome evaluations are pivotal for appraising the success or failure of oral rehabilitation, especially for patients with head and neck cancer (HNC) who have significant functional and psychological deficits [7]. LORQv3 is a specific questionnaire for cancer of the craniofacial region, which was developed in 2004 to comprehensively assess the multifaceted effects of prosthetic rehabilitation in these subjects. Various studies conducted using LORQv3 have included patients with malignancies in specific sites, such as the maxillary sinus [10,11]. The current study expands the scope by including patients with malignant lesions in the nasopharynx, maxillary sinus, oral cavity, nasal cavity with intracranial and infraorbital extensions, as well as the soft palate. In assessing the study cohort using LORQv3, improvements were observed in all domains from the preprosthetic phase to one month after rehabilitation. OHIP-14 is a tool that helps us assess the social and/or psychological impact of oral function. It helps in ascertaining self-reported dysfunction and discomfort caused by oral conditions [11]. Assessment using the OHIP-14 also showed significant improvement in all domains from the preprosthetic phase to one-month follow-up after rehabilitation, which is similar to the results observed in studies by Dholam et al. [12]. These improvements highlight the immediate and noticeable advantages of prosthetic intervention in terms of both psychological and functional aspects. During the six-month follow-up, all patients demonstrated continued improvement; however, for both the LORQv3 and OHIP-14 assessments, the percentage change was significantly smaller than the change seen between the preprosthetic phase and the one-month postrehabilitation follow-up. The initial restoration of oral function after a protracted period of dysfunction may have had a greater impact on patients than the subsequent incremental improvements seen between the one-month and six-month follow-up intervals, which could account for this attenuated improvement. This pattern can be explained through psychological adaptation theories, which suggest that people adjust to changes in health or function over time, making the initial improvements feel more salient than the later stabilization [13]. There were no declined respondents, as it was a questionnaire-based interview-type survey.

Functional recovery and psychological outcomes

It has been demonstrated that chewing disability resulting from the loss of natural teeth considerably lowers OHRQoL [14]. Oral cancer or its associated treatments often result in dysphagia [15]. Abdelfattah Mohamed and Kothayer ascertained a significant improvement in chewing ability and swallowing following obturator rehabilitation in patients with maxillary resection [16]. In the present study, prosthetic rehabilitation aids in improving chewing ability and promoting safe swallowing strategies, in line with studies conducted by Kalaignan and Ahmed [17] and Kreeft et al. [18]. One of the most impaired functions following head and neck surgery is speech due to surgical defects in sites like the palate, cheek, sinus, and tongue. A definite improvement in speech is observed in both first and second follow-ups after rehabilitation in this study, consistent with the findings of Yoshida et al. [19]. These findings demonstrate how important prosthetic intervention is to patients' functional recovery after treatment for oral cancer.

HNC treatment severely impairs esthetics, which adversely affects quality of life [10]. Regaining normal orofacial appearance after surgical resection can be challenging due to decreased retention, limited mouth opening, a lack of resilience in soft tissues, and widespread tissue defects [20]. A dramatic enhancement in scores related to orofacial appearance for these patients could be due to the fact that patients received prosthetic rehabilitation shortly after surgery, similar to previous studies [14,21]. A marked improvement in mouth opening was observed in these subjects after prosthetic rehabilitation, which helps prevent long-term functional issues, as reported by Rapidis et al. [22] and Yang et al. [23].

Notable improvements in a number of physical domains, such as decrease in physical pain (3.17 ± 0.65 → 1.13 ± 0.87 → 0.51 ± 0.75), improvement in their capacity to chew (3.39 ± 0.78 → 2.24 ± 0.60 → 1.54 ± 0.44), swallow (3.48 ± 0.76 → 1.96 ± 0.60 → 1.32 ± 0.41), and facial appearance (3.18 ± 0.88 → 2.09 ± 0.72 → 1.67 ± 0.74) were observed within the study cohort. These physical enhancements are especially significant because they have a direct impact on the patient's quality of life. Additionally, psychological well-being might have benefited from these improvements in oral function. The capacity to communicate, eat, and engage in social interactions without experiencing discomfort or embarrassment probably helped to lower anxiety and depression levels. Enhancements in facial appearance may have improved self-esteem and self-confidence. Collectively, these factors suggest that successful prosthetic rehabilitation not only addresses functional deficits but also plays an important role in restoring the comprehensive psychosocial health of these patients. The observed improvements in physical pain, swallowing, chewing, and appearance among the study participants were possibly facilitated by their enhanced psychological well-being, similar to studies by Reyes et al. [24] and Humphris and Ozakinci [25].

In a study by Rogers et al. [26], a cohort of 115 patients with a mean follow-up of 3.91 years reported a 94% improvement in OHRQoL in patients restored with implant prostheses, highlighting the efficacy of implant-supported rehabilitation in this population.

A high level of convergence in the evaluation of OHRQoL was indicated by a statistically meaningful positive correlation between the respective domains of the OHIP-14 and LORQv3 instruments. The validity and sensitivity of both instruments in capturing the multifaceted effects of oral rehabilitation, including functional, cosmetic, and psychosocial aspects, in patients receiving treatment for orofacial malignancies are highlighted by this domain-specific alignment. Given the robustness of this link, OHIP-14 and LORQv3 may be used in tandem or, in some therapeutic settings, interchangeably to offer a thorough assessment of treatment results. The usefulness of patient-reported outcome measures in directing and evaluating prosthodontic therapies in oncologic rehabilitation settings is further supported by these findings.

Strengths

In accordance with STROBE recommendations, the strengths of this study include the use of validated, patient-centered instruments, repeated measures over two follow-up periods, and clearly defined inclusion/exclusion criteria to minimize bias.

Limitations

This research was conducted at a single institution with a limited sample size (n = 44); therefore, the application of the study's results should be interpreted with caution. Increasing the sample size in subsequent research would provide greater statistical robustness and may yield more reliable and generalizable outcomes. Potential response bias from patients and interviewer bias, along with variability in tumor location and extent, may have influenced the outcome. Furthermore, it is difficult to evaluate the long-term sustainability of the observed results due to the brief follow-up period (one and six months).To strengthen the evidence base, subsequent studies may be conducted using prolonged follow-up intervals, such as five years, enabling a more comprehensive evaluation of the sustained effects of prosthetic rehabilitation on OHRQoL.

Generalizability

While the results are most directly applicable to adult patients undergoing prosthodontic rehabilitation following oral neoplasm treatment, the findings can be cautiously extrapolated to similar populations requiring oral rehabilitation. Future site-specific, multicenter studies with larger sample sizes could enhance generalizability.

Future prospects

Clinical evaluations alone might not always reveal the functional, emotional, and social difficulties that patients undergoing oral rehabilitation face. By employing these questionnaires, clinicians can customize treatment regimens to meet the unique requirements and priorities of patients by recognizing common problems like speaking, chewing, appearance, or social interaction challenges. In addition to encouraging innovation in prosthesis design, the input gathered from these surveys helps enhance comfort, functionality, and appearance.

Data from this research can support healthcare policies and budget allocation. The consistent application of LORQ and OHIP also facilitates the tracking of patient satisfaction and recovery over the long term, creating standards for assessing treatment effectiveness.

## Conclusions

Patients treated for HNC show marked improvement in OHRQoL following prosthetic rehabilitation. Assessments using validated instruments, such as OHIP-14 and LORQv3, reveal a significant enhancement in functional performance, esthetic satisfaction, and psychosocial well-being within the first month after rehabilitation. Although further gains are observed up to six months, the pace of improvement slows, suggesting a phase of stabilization. All evaluated domains demonstrated statistically significant progress, except for mandibular prosthesis satisfaction, indicating a scope for refinement in this area. These findings emphasize that timely prosthetic rehabilitation not only restores essential oral functions but also alleviates psychological distress and social challenges, underscoring the importance of sustained multidisciplinary support throughout the recovery period. The data as a whole demonstrate the significant and early benefits of prosthetic rehabilitation on OHRQoL and support the use of OHIP-14 and LORQv3 as reliable instruments for outcome assessment in this patient group.

## Appendices

**Table 6:** OHIP-14 questionnaire OHIP-14: Oral Health Impact Profile 14

Sl. no.	Question	Never (0)	Hardly ever (1)	Sometimes (2)	Often (3)	Very often (4)
1	Have you had trouble pronouncing any words because of problems with your teeth, mouth, or dentures?	☐	☐	☐	☐	☐
2	Have you felt that your sense of taste has worsened because of problems with your teeth, mouth, or dentures?	☐	☐	☐	☐	☐
3	Have you had painful aching in your mouth?	☐	☐	☐	☐	☐
4	Have you found it uncomfortable to eat any foods because of problems with your teeth, mouth, or dentures?	☐	☐	☐	☐	☐
5	Have you been self-conscious because of your teeth, mouth, or dentures?	☐	☐	☐	☐	☐
6	Have you felt tense because of problems with your teeth, mouth, or dentures?	☐	☐	☐	☐	☐
7	Has your diet been unsatisfactory because of problems with your teeth, mouth, or dentures?	☐	☐	☐	☐	☐
8	Have you had to interrupt meals because of problems with your teeth, mouth, or dentures?	☐	☐	☐	☐	☐
9	Have you found it difficult to relax because of problems with your teeth, mouth, or dentures?	☐	☐	☐	☐	☐
10	Have you been a bit embarrassed because of problems with your teeth, mouth, or dentures?	☐	☐	☐	☐	☐
11	Have you been a bit irritable with other people because of problems with your teeth, mouth, or dentures?	☐	☐	☐	☐	☐
12	Have you had difficulty doing your usual jobs because of problems with your teeth, mouth, or dentures?	☐	☐	☐	☐	☐
13	Have you felt that life in general was less satisfying because of problems with your teeth, mouth, or dentures?	☐	☐	☐	☐	☐
14	Have you been totally unable to function because of problems with your teeth, mouth, or dentures?	☐	☐	☐	☐	☐

**Table 7:** Liverpool oral rehabilitation questionnaire version 3

Sl. no.	Question	Never (1)	Sometimes (2)	Often (3)	Always (4)
Section 1: Oral function, orofacial appearance, and social interaction					
1.	Did you experience difficulty with chewing?	☐	☐	☐	☐
2.	Did you have pain when you chew?	☐	☐	☐	☐
3.	Did you experience difficulty with swallowing solids?	☐	☐	☐	☐
4.	Did you experience difficulty with swallowing liquids?	☐	☐	☐	☐
5.	Did food particles collect under your tongue?	☐	☐	☐	☐
6.	Did food particles stick to your palate?	☐	☐	☐	☐
7.	Did food particles stick inside your cheeks?	☐	☐	☐	☐
8.	Did you have mouth dryness?	☐	☐	☐	☐
9.	Did you have problems with drooling?	☐	☐	☐	☐
10.	Did you experience problems with speech?	☐	☐	☐	☐
11.	Were you upset by your facial appearance?	☐	☐	☐	☐
12.	Were you upset by the appearance of your mouth?	☐	☐	☐	☐
13.	Were you upset by the appearance of your lips?	☐	☐	☐	☐
14.	Were you upset by the appearance of your teeth?	☐	☐	☐	☐
15.	Did your chewing ability affect your social life?	☐	☐	☐	☐
16.	Did your chewing ability influence your choice of foods?	☐	☐	☐	☐
17.	Did you experience difficulty opening your mouth?	☐	☐	☐	☐
Section 2: Prevalence of natural teeth, dentures, and implant-retained teeth					
18.	Do you have any natural teeth in the upper jaw?	☐ Yes		☐No	
19.	Do you have any natural teeth in the lower jaw (or any in the upper/lower)?	☐ Yes		☐No	
Section 3: Prostheses and patient satisfaction					
20.	Were you embarrassed about conversing because of your dentures/implant retained teeth?	☐	☐	☐	☐
21.	Did you refuse dinner invitations because of embarrassment about your dentures/implant retained teeth?	☐	☐	☐	☐
22.	Did you feel loss of self-confidence because of embarrassment about your dentures/implant retained teeth?	☐	☐	☐	☐
23.	Did you find it difficult to open your mouth because of your dentures/implant retained teeth?	☐	☐	☐	☐
Section 2: Prevalence of natural teeth, dentures, and implant-retained teeth					
24.	Do you have an upper denture?	☐Yes ☐No		☐Yes ☐No	
25.	Do you have upper implant retained teeth (or upper denture/implant)?	☐Yes ☐No		☐Yes ☐No	
Section 3: Prostheses and patient satisfaction					
26.	Were you dissatisfied with your upper denture/implant retained teeth?	☐	☐	☐	☐
27.	Did your upper denture/implant retained teeth cause soreness or ulceration of the gum?	☐	☐	☐	☐
28.	Did you find food particles collecting under your upper denture/implant retained teeth?	☐	☐	☐	☐
29.	Did you take out your upper denture/implant-retained teeth for eating?	☐	☐	☐	☐
30.	Did you feel insecure with your upper denture/implant retained teeth?^*^	☐	☐	☐	☐
31.	Were you worried that your upper denture/implant retained teeth might fall out?	☐	☐	☐	☐
Section 2: Prevalence of natural teeth, dentures, and implant-retained teeth					
32.	Do you have a lower denture?	☐Yes ☐No		☐Yes ☐No	
33.	Do you have lower implant retained teeth (or lower denture/implant)?	☐Yes ☐No		☐Yes ☐No	
Section 3: Prostheses and patient satisfaction					
34.	Were you dissatisfied with your lower denture/implant retained teeth?	☐	☐	☐	☐
35.	Did you lower denture/implant retained teeth cause soreness or ulceration of the gum?	☐	☐	☐	☐
36.	Did you find food particles collecting under your lower denture/implant retained teeth?	☐	☐	☐	☐
37.	Did you take out your lower denture/implant retained teeth for eating?	☐	☐	☐	☐
38.	Did you feel insecure with your lower denture/implant retained teeth?	☐	☐	☐	☐
39.	Were you worried that your lower denture/implant retained teeth might fall out?	☐	☐	☐	☐
